# Global Health Priority Box—Proactive Pandemic Preparedness

**DOI:** 10.1021/acsinfecdis.4c00700

**Published:** 2024-11-03

**Authors:** Anna Adam, Dominique Besson, Rob Bryant, Sarah Rees, Paul A. Willis, Jeremy N. Burrows, Rob Hooft van Huisjduijnen, Benoît Laleu, Larry Norton, Stacie Canan, Natalie Hawryluk, Dale Robinson, Mike Palmer, Kirandeep Kaur Samby

**Affiliations:** 1 MMV Medicines for Malaria Venture, 1215 Geneva, Switzerland; 2 Innovative Vector Control Consortium, L3 5QA Liverpool, United Kingdom; 3 Bristol Myers Squibb, California 92121, San Diego, United States of America; 4 Brychem/Agranova, BR6 9AP Kent, United Kingdom

**Keywords:** Resistant malaria, open access box, drug discovery, zoonotic diseases, vector control, neglected diseases

## Abstract

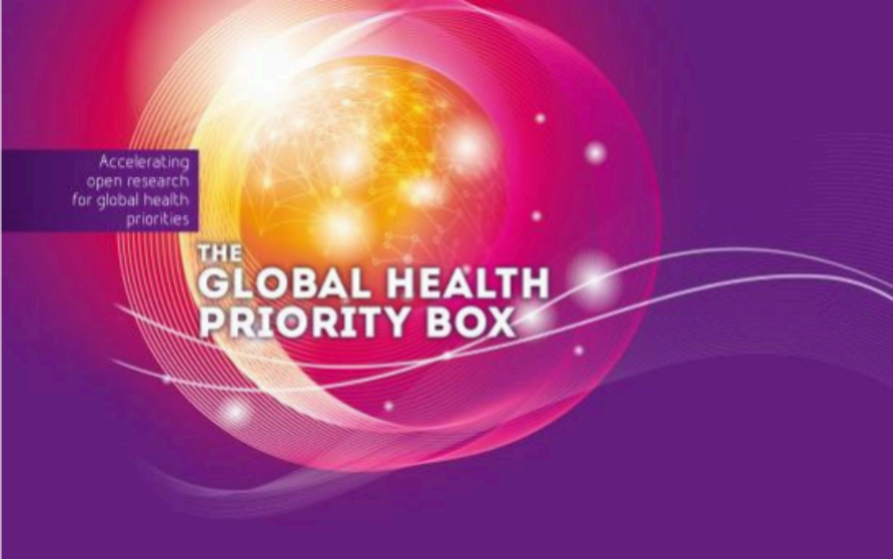

The coronavirus pandemic outbreak of 2019 highlighted the critical importance of preparedness for current and future public health threats (https://www.mmv.org/mmv-open/global-health-priority-box/about-global-health-priority-box). While the main attention for the past few years has been on COVID-19 research, this focus has reduced global resources on research in other areas, including malaria and neglected tropical diseases (NTDs). Such a shift in focus puts at risk the hard-earned progress in global health achieved over the past two decades (https://www.who.int/news-room/spotlight/10-global-health-issues-to-track-in-2021). To address the urgent need for new drugs to combat drug-resistant malaria, emerging zoonotic diseases, and vector control, Medicines for Malaria Venture (MMV) and Innovative Vector Control Consortium (IVCC) assembled a collection of 240 compounds and, in August 2022, launched the Global Health Priority Box (GHPB). This collection of compounds has confirmed activity against emerging pathogens or vectors and is available free of charge. This valuable tool enables researchers worldwide to build on each other’s work and save precious time and resources by providing a starting point for the further development of treatments and insecticides. Furthermore, this open access box aligns with two of the many priorities outlined by the World Health Organization (WHO) (https://www.who.int/news-room/spotlight/10-global-health-issues-to-track-in-2021).

## Introduction

Neglected tropical diseases (NTDs) are a diverse group of infectious diseases that are mainly prevalent in tropical and subtropical countries, and it is estimated that more than 1.6 billion people are affected by at least one of these diseases every year.^3^ NTDs have a significant impact on impoverished communities, both in terms of the number of affected individuals and the economic losses incurred, leading to staggering losses of billions of dollars.^4^ NTDs are caused by a variety of pathogens including viruses, bacteria, parasites, fungi, and vectors. Unfortunately, the lack of research and development for these diseases with limited to no commercial value, coupled with the emergence of resistant pathogens, has resulted in inadequate treatment options for many of these diseases.^5^ Recognizing the enormity of the problem, in 2021, the World Health Organization (WHO) created a roadmap to end NTDs by 2030 and prioritized 20 neglected diseases (Table 1) that have a devastating impact on impoverished communities.^3^ Additionally, the WHO identified 10 global health issues and priorities to address in the future, particularly in response to the global pandemic.^2^

**Table 1:** List of WHO Prioritized Neglected Diseases

NTDs include: Buruli ulcer; Chagas disease; dengue and chikungunya; dracunculiasis; echinococcosis; foodborne trematodiases; human African trypanosomiasis; leishmaniasis; leprosy; lymphatic filariasis; mycetoma, chromoblastomycosis and other deep mycoses; onchocerciasis; rabies; scabies and other ectoparasitoses; schistosomiasis; soil-transmitted helminthiases; snakebite envenoming; taeniasis and cysticercosis; trachoma; and yaws.

To address the challenges associated with NTDs and malaria, the Global Health Priority Box (GHPB) was developed through a collaborative effort between Medicines for Malaria Venture (MMV) and the Innovative Vector Control Consortium (IVCC) and includes global health compounds donated by Bristol Myers Squibb (BMS). This open-access box is intended to provide researchers with verified starting points to expedite the development of treatments and insecticides/endectocides and address two of the several priorities established by the World Health Organization in late 2021, namely drug resistance and communicable diseases.^2^ The GHPB consists of three 96-well plates, with the compounds in each plate targeting different aspects of malaria and NTDs: drug-resistant malaria, zoonotic and neglected diseases, and vector control.

## Multidrug Resistant Malaria

Despite ongoing efforts to control and ultimately eradicate malaria, there has been a steady rise in cases since 2016.^6^ The World Malaria Report estimates that in 2022, there were over 249 million cases of malaria in 85 countries where the disease is endemic, leading to over 608,000 deaths in 2022. This represents an increase of 5 million cases from the previous year and over 19 million more cases since 2015.^7^ The rising number of cases could be attributed to a variety of factors, including the recent COVID-19 pandemic outbreak, which occurred between 2019 and 2020,^6^ as well as emerging insecticide resistance impacting the effectiveness of long-lasting insecticide treated nets.^8^

Partial resistance to artemisinin in K13 mutant parasites endangers the artemisinin combination therapies (ACTs) widely used in the field as a higher number of parasites surviving the exposure to the artemisinin component are exposed to the longer lasting partner drug and thus are more likely to develop partner drug resistance.^9^ Clinical ACT failure, resulting from partner drug resistance, has already been reported in Southeast Asia^10^ with certain ACTs, and subsequent treatment failures in the Greater Mekong subregion have raised concerns of losing the currently only highly effective treatment available to treat malaria.^11^ Currently, artemether-lumefantrine and pyronaridine-artesunate remain efficacious ACTs in all geographies.^12,13^

Moreover, recent reports show a concerning trend in the spread of *Anopheles stephensi*, historically considered as an urban Asian malaria vector, in Africa. This invasive species has effectively made its way to Ethiopia,^14^ Somalia,^15^ Sudan^16^ or Kenya^17^ and is capable of transmitting both *Plasmodium falciparum* and *Plasmodium vivax*,^18^ with the latter capable of forming dormant liver stages that can reactivate and cause new symptomatic blood stage infections.^18^ If this vector becomes widespread, it could significantly increase transmission and cases of malaria infection in Africa. Additional concerns are that this invasive species thrives in urban settings and has been reported to be resistant to several classes of insecticides.^19^

In response to these threats, over the past decade, interest in antimalarial drug development has grown^6^ and resulted in 35 candidate drugs being delivered by MMV with its Discovery partners, of which 21 are in preclinical or clinical development.^20,21^ While this is an encouraging development, it is important to note that many of these candidates may fail to progress in clinical development due to inadequate pharmacokinetics, inadequate efficacy, safety concerns, chemical manufacturing risks, or parasite resistance. Therefore, the need for safe molecules with novel mechanisms of actions^22^ clearing asexual blood stage and preventing transmission to human or to mosquito vector is high.

Due to advancement in robotic automation and liquid handling, high throughput screening (HTS) allows testing of very large compound libraries.^23^ For the past 15 years, phenotypic screening approaches have typically been used to identify new hit series that have been optimized to antimalarial drug candidates.^24,25^ One of the limitations of phenotypic screening, however, is the lack of information about a compound’s mode of action and its off-target effects in the absence of biochemical or cellular target deconvolution assays; the mode of action, when known, influences a compound’s optimization and safety evaluation.^26^ Knowing the mode of action of a compound is important because series with widely exemplified and well worked mechanisms of resistance (MoR) and action may be down-prioritized versus novel alternatives. Furthermore, knowledge of the biological target allows selectivity testing to be performed with the human and preclinical species’ orthologues to maximize the safety profile of a candidate drug.

Over 10 million compounds have now been tested against asexual blood-stage parasites,^26^ resulting in a number of hits that have been further profiled, optimized, and developed into drug candidates such as Sutidiazine (ZY 19489),^27^ Cabamaquine (M5717)^28^ and Ganaplacide (KAF156).^29^ Due to the rising threat of drug resistance in asexual blood stage parasites, additional sets of compounds were screened to identify inhibitors targeting other parasite lifecycle stages such as liver and transmission phases to mitigate the transmission of resistance.

## Vectors

Vector-borne diseases (VBDs) are infections caused by pathogens that are transmitted by vectors.^30^ In 2020, WHO estimated that more than 17% of all infectious diseases caused by parasites, bacteria or viruses are transmitted via vectors and result in more than 700,000 deaths annually.^24^ The number of annual deaths has risen since 2020, and malaria infection accounts for over 600,000 annual deaths.^7^ In NTD transmission, the arthropod vectors play a crucial role, transmitting pathogens that cause diseases such as Chagas disease, Dengue, leishmaniasis, Japanese encephalitis, lymphatic filariasis, and yellow fever. These diseases not only pose a threat to over 80% of the global population^31^ but also have a major impact on the poorest populations living in the tropical and subtropical regions, where disease control resources and health systems can be challenged. It is also estimated that more than half the world’s population lives in areas where there are 2 or more VBDs prevalent.^31^

Many of these vector-borne diseases also fall under the category of NTDs.^32^ Unfortunately, due to a lack of understanding of the burden imposed by NTDs in the past, research and medicines to treat these diseases have suffered from a lack of prioritization and investment.

Vector control programs have been responsible for reduction of Chagas disease in South America^33^ or near elimination of river blindness in large parts of West Africa.^34^ Long lasting insecticidal nets (LLINs) and Indoor Residual Spraying (IRS) are two core vector control interventions used widely for malaria prevention, with great effect.^35^

The latest World Malaria report^6^ has highlighted emerging threats as malaria vectors and parasites rapidly evolve, rendering current preventive and treatment tools less effective. For instance, there is widespread resistance to pyrethroids,^36^ the primary class of insecticides used in insecticide-treated nets. Although insecticide treated bed nets with a new mode of action that overcome current resistance mechanisms are being introduced,^37,38^ the emergence of resistance underscores the continuous need for the development of effective tools to combat vector-borne diseases. While the use of certain classes of insecticides has decreased, either due to resistance or harmful effects, as seen with pyrethroids, organochlorines and organophosphates, these insecticides are still used to varying degrees depending on the type of intervention, the region, and the specific vectors they are targeting.^39^

## Neglected and Zoonotic Diseases with Risk of Drug Resistance

WHO defines zoonotic diseases as diseases that are naturally transmitted from vertebrate animals to humans and vice-versa.^40^ Studies show that more than half of pathogens capable of infecting humans are zoonotic^41^ and originate from wildlife reservoirs.^42^ Environmental and climate changes, coupled with shifts in human living patterns (such as encroachment into wildlife habitats, movement of wildlife from degraded areas to urban settings, and mass gatherings of people), present new challenges to global health, impacting people, animals, and ecosystems, and create fertile ground for the emergence and spill-over of new infectious diseases.^43^

The emergence of new pathogens transmissible from animals to humans presents a silent yet potent threat of a new deadly disease outbreak. The recent COVID-19 pandemic exemplifies how easily animal pathogens can adapt and infect humans causing fatal diseases that affect billions of people worldwide.^44^ It underscores the importance of preparedness for existing and emerging threats.

Emerging epidemics and pandemics present ongoing threats, and the key to effectively combating these pathogens lies in preparedness and early drug discovery to deliver prophylactic and treatment options long before the emergence of such deadly pathogens. Testing compounds early on a wide range of pathogens with pandemic potential would enable scientists to stay ahead, enabling the delivery of well-characterized drugs ready for further development and roll out, when needed, for maximal public health benefit.

## Composition of Global Health Priority Box

The Global Health Priority Box (GHPB) is a diverse collection of 240 compounds designed to facilitate drug discovery research in infectious diseases and provide antimalarial compounds for target deconvolution.

The GHPB comprises three plates, each focusing on different areas of pathogens that cause infectious diseases.

**Figure 1:**
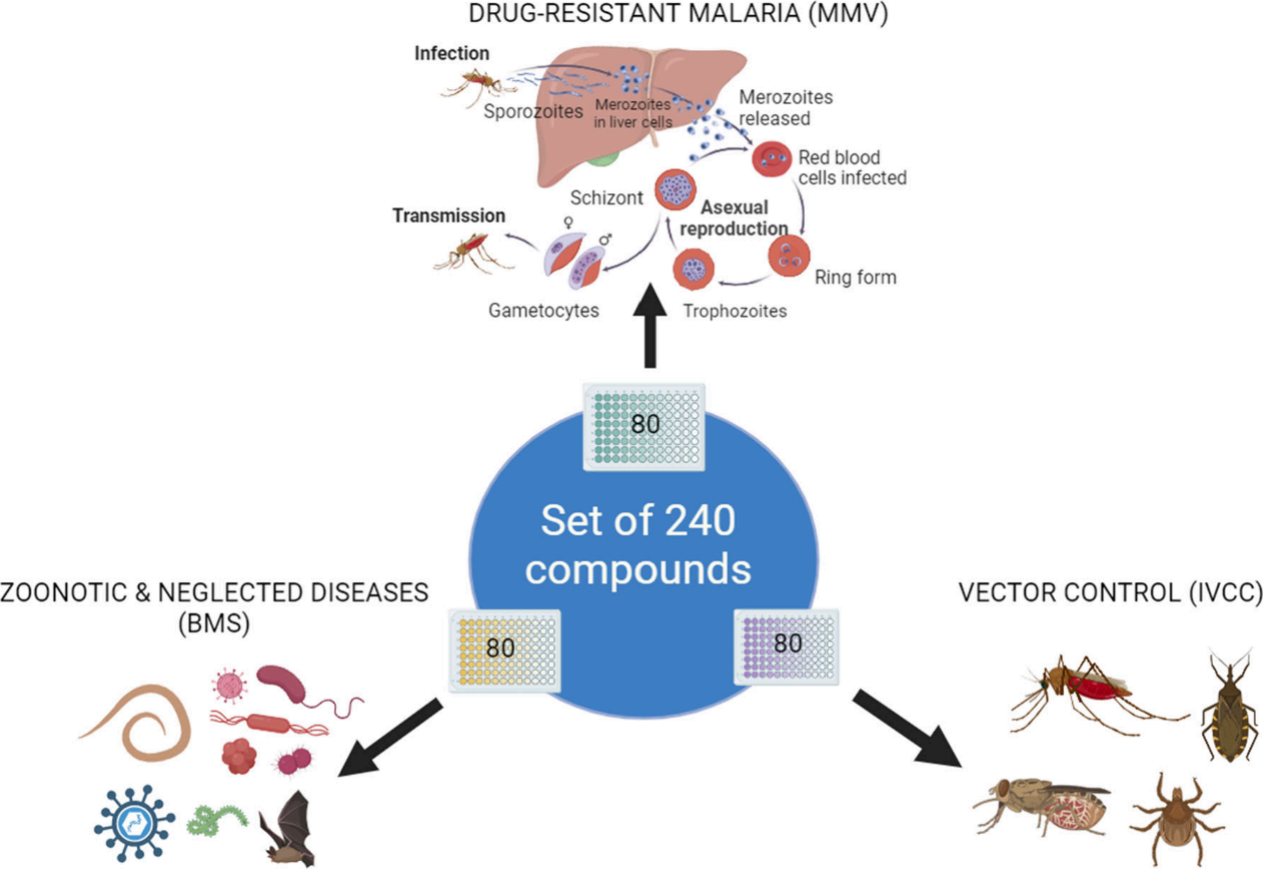
Constituents of the Global Health Priority Box.

**The Antimalarials plate** contains 80 compounds specifically chosen for their confirmed activity against drug-resistant malaria. Additionally, it includes novel compounds with unknown modes of action (MoA) to stimulate further investigations into new validated targets and, thus, support drug discovery efforts. **The Zoonotic& Neglected Diseases plate**, consisting of 80 compounds, is derived from a compound library generously donated by Bristol-Myers Squibb (former Celgene Global Health). These compounds are selected for their potential efficacy against NTDs, zoonotic diseases, and microbial diseases with a risk of drug resistance such as malaria, tuberculosis, Chagas disease, Human African trypanosomiasis, or cryptosporidiosis. **A Vector Control - Endectocide/Insecticide plate** also contains 80 compounds and was assembled in collaboration with the **Innovative Vector Control Consortium (IVCC)** to stimulate endectocide drug discovery and target additional insect vectors by distributing a diverse set of known agrochemicals, insecticides and endectocides (Figure 1).

## Methods (Constitution and Selection of GHPB Compounds)

### Vector Control Plate

**Figure 2:**
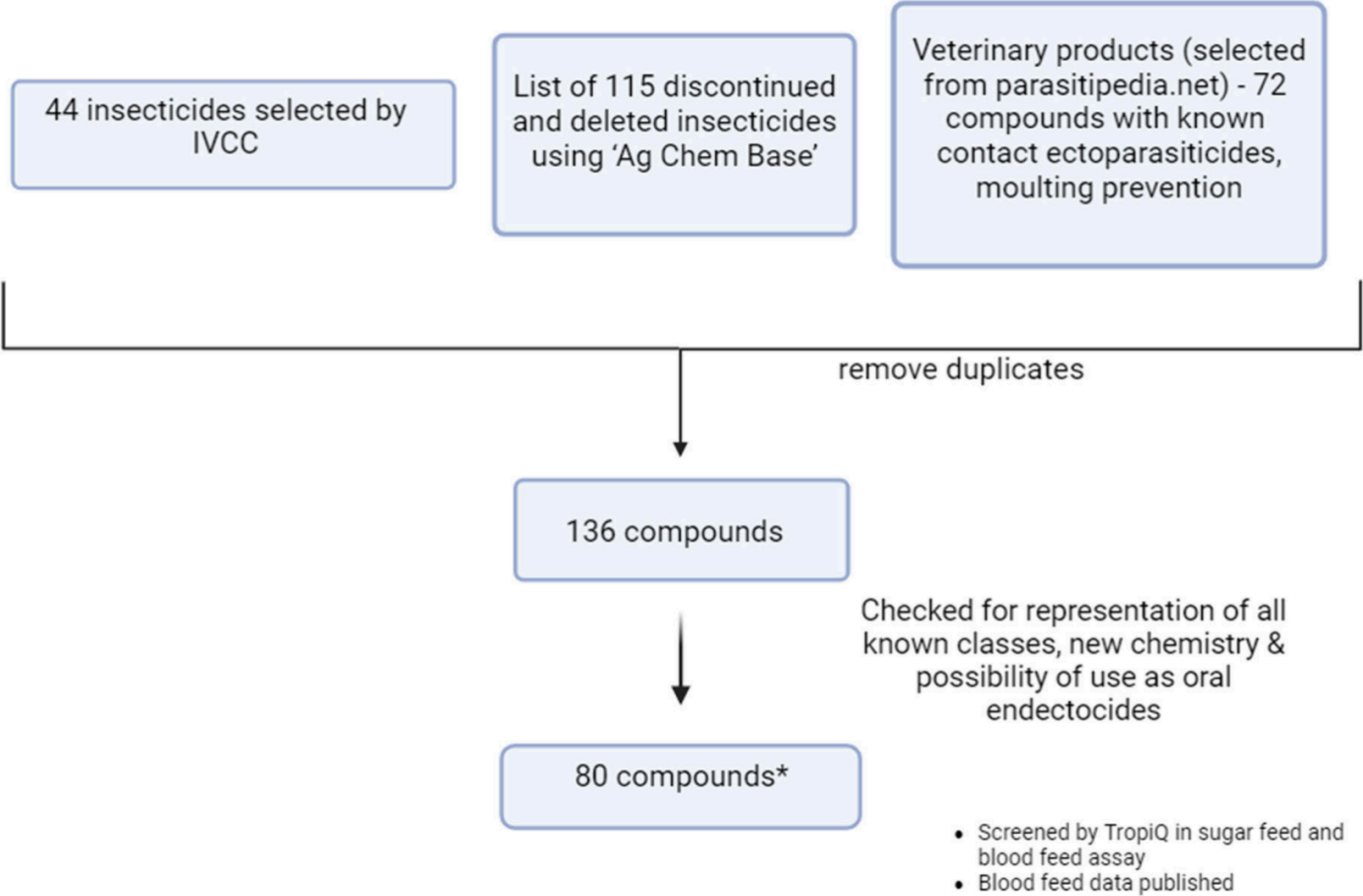
Vector plate selection workflow.

Repositioning existing insecticides or agrochemicals originally developed for nonvector control purposes can be a cost-effective and time-efficient strategy for developing vector control products. In order to provide the scientific community with a comprehensive set of compounds for this purpose, the MMV compiled a list of 231 compounds, consisting of 159 insecticides and 72 veterinary products and agrochemicals. The compilation was based on data from Ag Chem Base, a database containing information on developmental and commercial crop protection compounds and biological agents,^45^ and parasitipedia.net.^46^ By triaging compounds with known ectoparasiticidal activity, an additional 44 insecticides were selected by IVCC. After removal of duplicates, a list of 136 compounds was further narrowed down based on novelty and the possibility of using these compounds as attractive sugar bait or oral endectocides (Figure 2).

Initially, the intention was to exclude compounds that had demonstrated resistance in the field against malaria vectors. However, it was decided to include some examples of these scaffolds in order to explore their activity against other disease vectors, such as those responsible for leishmaniasis and dengue.

**Figure 3:**
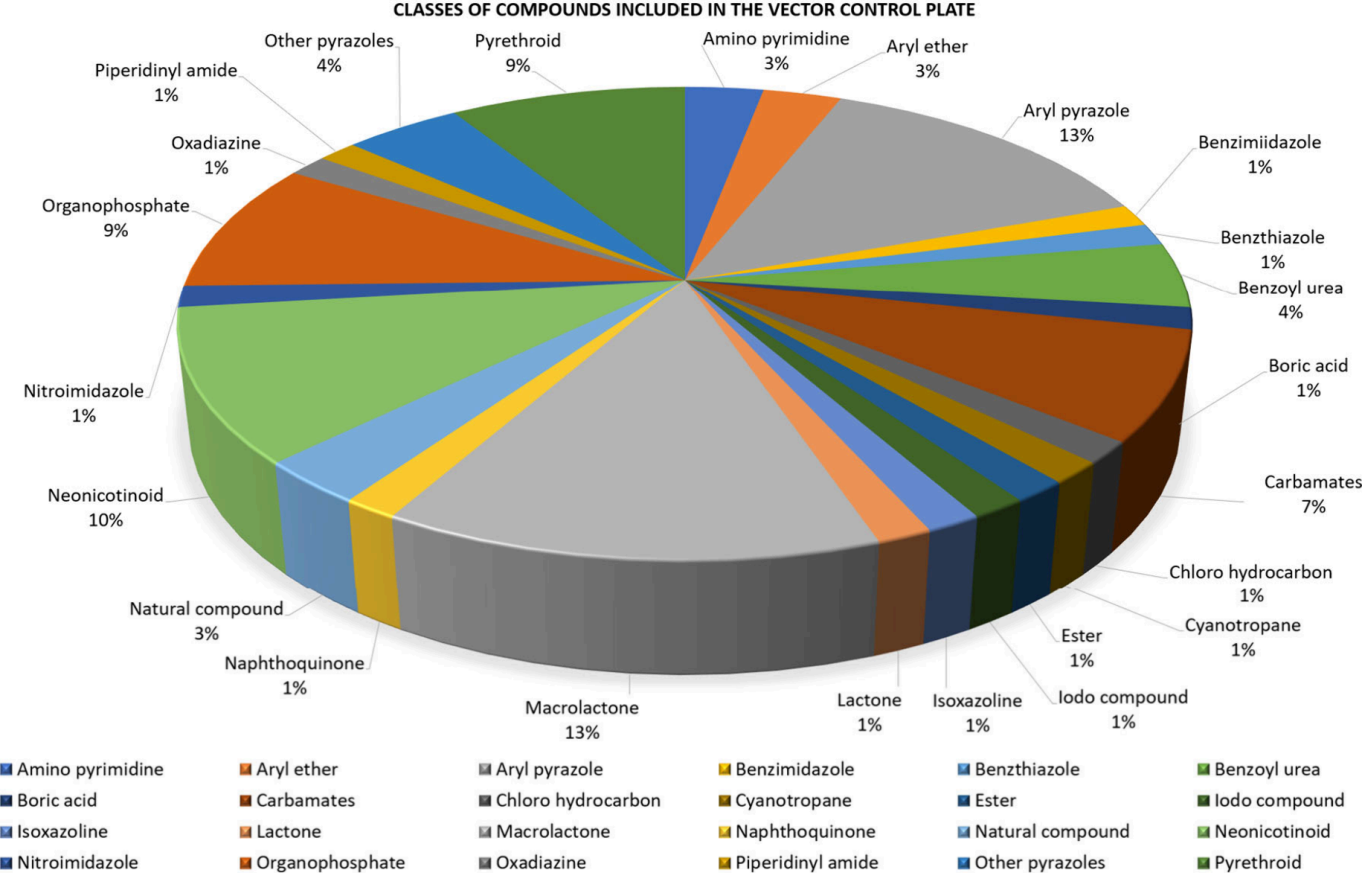
Classes of compounds included in the Vector Control Plate.

The shortlisted compounds were procured or synthesized by TCGLS, a contract research organization based in India. Figure 3 shows the diversity of chemotypes included in the vector control set.

### Zoonotic and Neglected Disease Plate

**Figure 4:**
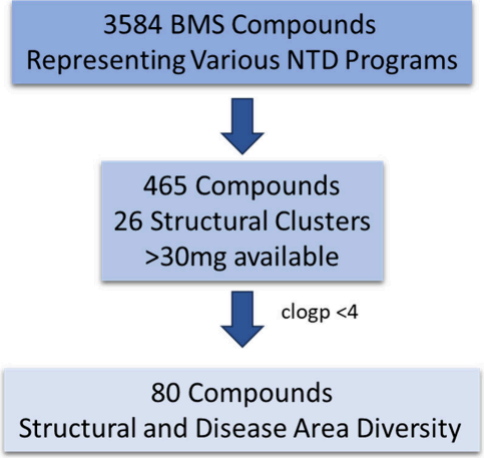
Zoonotic disease compound selection workflow

An initial set of 3584 compounds synthesized for various NTD programs were provided by the scientists at BMS. From these 3584 compounds, a selected set of 465 compounds representing 26 structural clusters and spanning across various disease areas as well as availability of sufficient sample quantities (>30 mg powder) were used for further triaging. Screening >100 compounds in endemic countries where screening capacity is limited by resource availability is not feasible. Therefore, to address this, compounds were further triaged based on scaffold diversity among the disease areas along with desirable physicochemical properties, ensuring a clogP value of less than 4 (Figure 4). Additionally, chemotypes represented in previous Open Access boxes were excluded.

**Figure 5:**
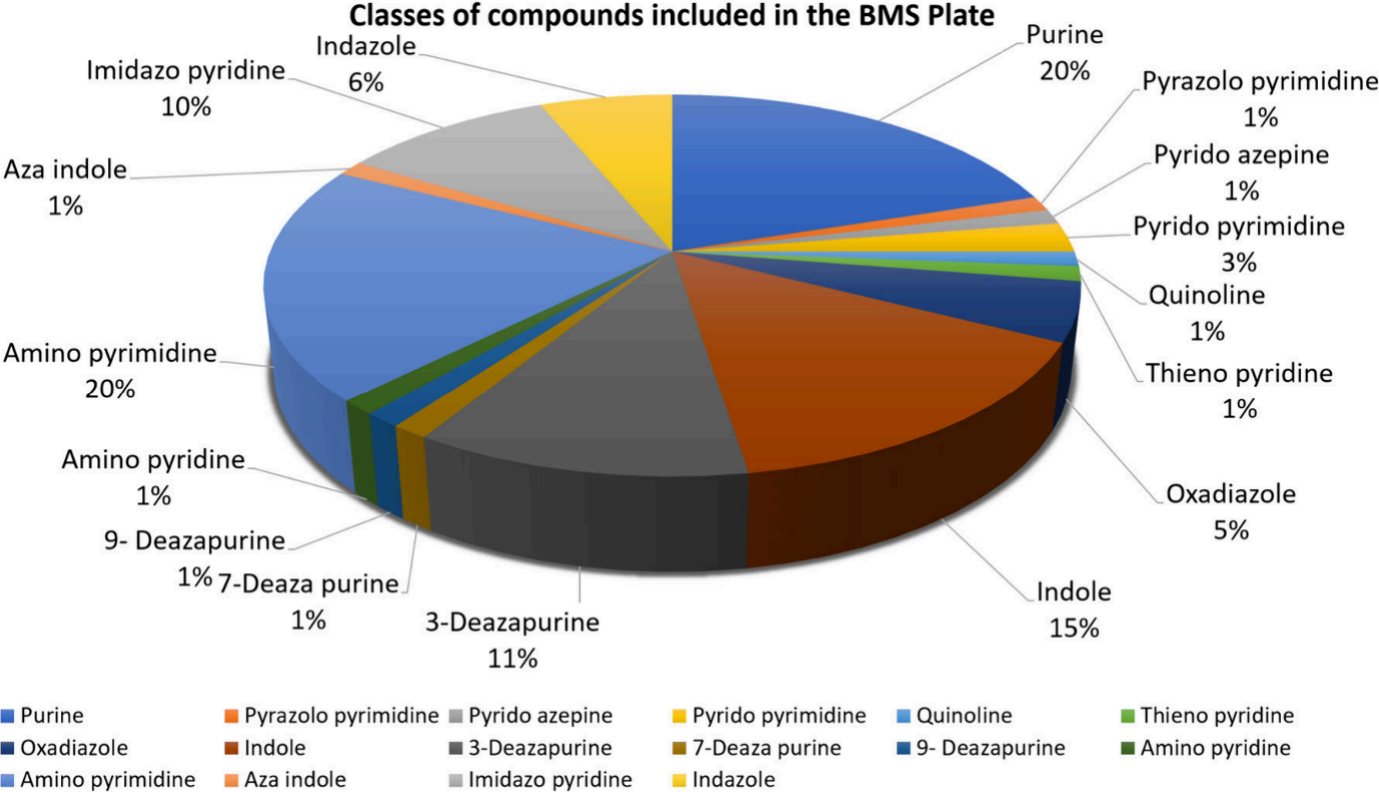
Classes of compound included in the Zoonotic disease plate.

**Figure 6:**
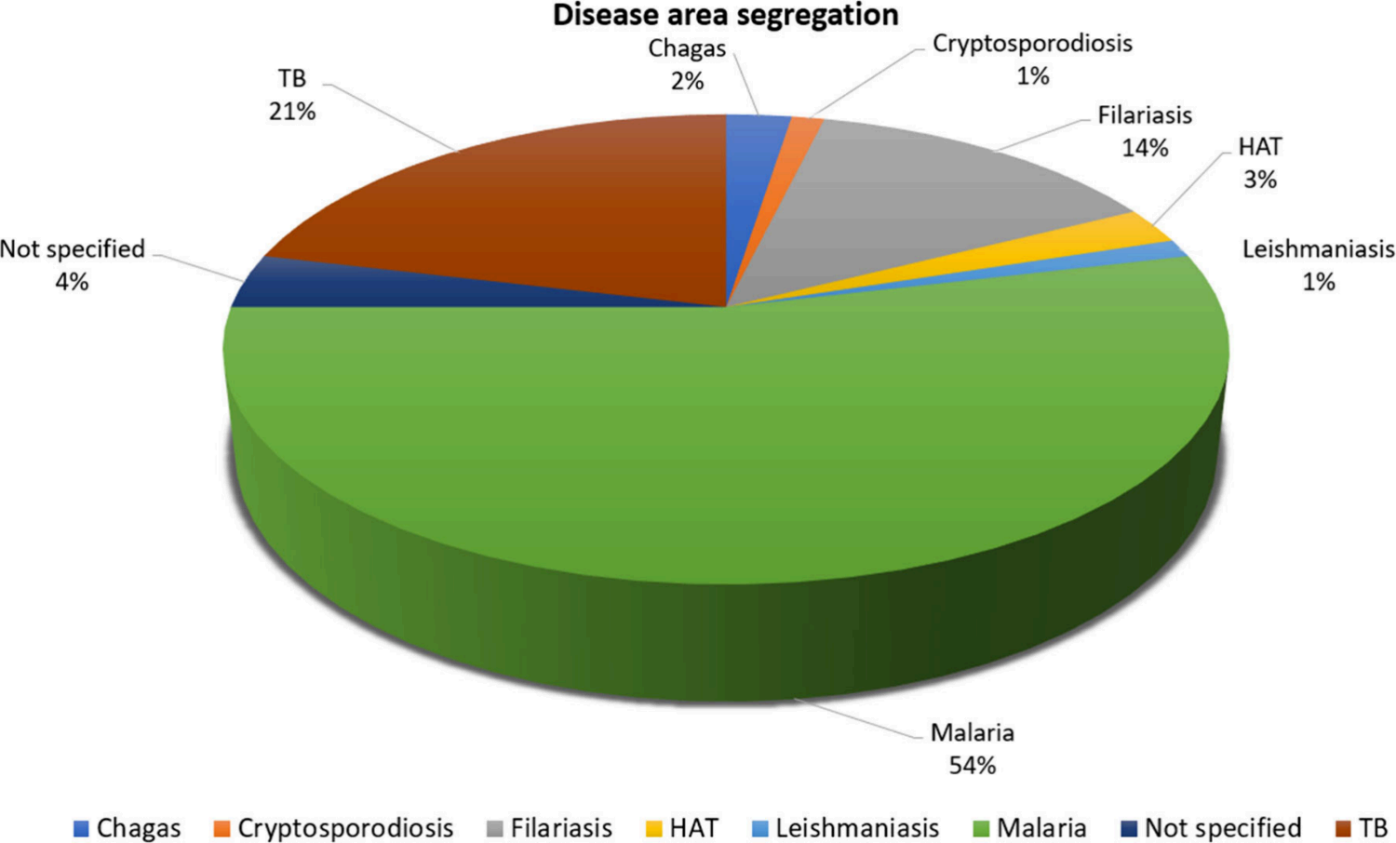
Disease area segregation.

A final list of 80 compounds was selected (Figure 5) representing 16 structural clusters and various disease areas (Figure 6). The bias toward malaria can be explained by the higher number of structural analogues available from several lead optimization programs performed around 5 main clusters (purine, imidazopyridine, indazole, aminopyrimidine, aminopyridine). We anticipate that some of these compound classes may have cross-disease activity.

### Drug Resistant Malaria Plate or Set

Due to the extensive amount of chemistry that has been explored through phenotypic screening in the pursuit of potent “hits”, finding novel compound libraries to screen is becoming increasingly difficult.^47^

Screening compound libraries in phenotypic assays has identified hits with known MoR/MoA; however, such low priority mechanisms have only been deconvoluted after considerable resource investment based on assay availability. To remedy this, MMV adopted a workflow that helps to rapidly prioritize the most interesting compounds and eliminate low priority actives at the stage of scaffold selection for hit validation.

This same workflow has been used during the selection of compounds for the drug resistant malaria plate. The majority of compounds do not have a known mechanism of resistance (MoR) based on cross-resistance studies with selected resistant cell lines. Since these compound sets are mainly requested by biologists to identify new starting points for drug discovery programs or as a chemical biology tool for target identification and validation, the associated information provides researchers with compounds that have confirmed antiplasmodial activity and knowledge of any prior screening data. This set can be used for target deconvolution using various techniques or the initiation of drug discovery programs. Based on experience with data generated by screening previous open access boxes, its cross screening is expected to provide interesting chemical matter to work on, especially against other apicomplexans.

A screening cascade for target deconvolution or identification of mechanisms of resistance was employed for the selection of compounds. We selected 125,000 compounds with reported *Plasmodium falciparum* asexual blood-stage (*Pf* ABS) activity from the ChEMBL database and removed known drugs and compounds with *Pf* ABS IC_50_ values greater than 10 μM. Chemotypes previously explored in previous open access boxes were also excluded. To expand the set, compounds were selected from a previous screen of more than 500,000 compounds against *Plasmodium berghei*—murine liver-stage parasites—in which 681 validated hits with efficacy at sub-micromolar concentrations against hepatic schizonts (TCP4) were identified.^48^ In addition, compounds were added from the ∼70,000 compound diversity library in which 17 compounds with transmission-blocking activity (TCP 5) were identified.^49^

**Figure 7:**
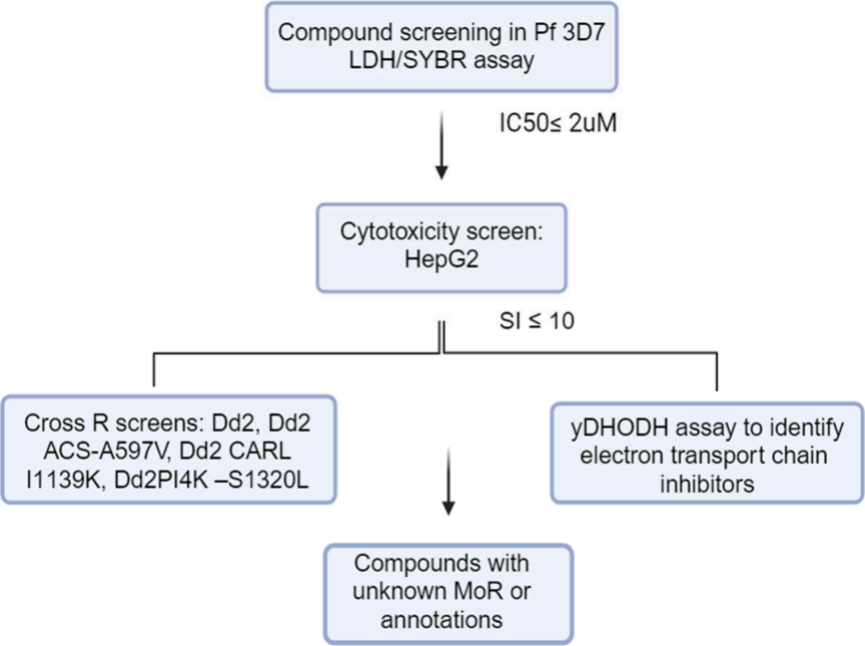
Malaria plate selection workflow.

Using StarDrop^50^ and input from medicinal chemists, we further narrowed down the selection to 620 compounds, including compounds with liver stage activity. These compounds were then subjected to primary screening using *Pf* 3D7 LDH and SYBR green assays. Only 114 compounds met the criteria of ABS activity less than 2 μM from the primary screens and exhibited low cytotoxicity with a selectivity index of 10 or higher. The compounds were further narrowed down by screening against several resistance panels to flag known mode of actions or mechanism of resistance (CARL, ACS, PI4K, DHODH, bc1). The multidrug resistant Dd2 strain was used as starting point in the following panel of genetically engineered strains: Dd2 CARL-I1139K, Carl, Dd2 ACS-A597V, and Dd2 PI4K-S1320L. The yeast DHODH assay was used to identify electron transport chain inhibitors targeting either bc1 or DHODH. Moreover, pH fingerprint assays enabled identification of *Pf*ATP4,^51^*Pf*FNT, the V-type H+ ATPase, and the acid-loading Cl- transport pathway (of unknown molecular identity) (Figure 7). For recent (2020–2022) libraries, compounds with selectivity index (SI) greater than 10 in a cytotoxicity assay using HepG2 cells were directly screened in the barcoded resistome pool, which consists of approximately 50 parasite strains, each containing a mutation in a given drug target gene that confers resistance. The final list consists of 80 compounds, including 2 gametocytocidal compounds without ABS activity. The drug-resistant malaria plate comprises novel scaffolds and compounds identified through literature reviews and ongoing library screenings conducted at MMV. Careful measures were taken to ensure that none of the compounds included in earlier collections are part of this set.

## Composition of the Global Health Priority Box

Based on feedback and demand from biological partners, the GHPB has been developed to provide three small, focused sets of 80 compounds (240 compounds in total), each with a specific focus. Within each of the three 96-well plates, 16 wells are available for appropriate positive and negative controls of the biological assay that will be conducted.

The compounds are provided as thin films, and the quantity corresponds to 10 mM stock when solubilized with 10 μL of DMSO. The reconstituted plates can be used as mother plates to create several daughter plates of the required concentration. After an initial dilution in 100% DMSO to preserve the solubility of the compounds at a high concentration, MMV recommends further dilutions to be performed in the reaction/assay buffer to lower the DMSO concentration in the assay as much as possible. This standardized set format allows researchers to compare results between laboratories^52^ and is suitable for automated and manual sample testing, allowing its use by scientists in Low and Middle-Income countries.

Near neighbors of any actives from the compound set identified by the recipients can be requested from the MMVOpen Team and will be resupplied when this is possible.

The GHPB is available free of charge from MMV upon request (https://www.mmv.org/mmv-open/global-health-priority-box/about-global-health-priority-box).

The details of the entire compound set are in the Supporting Information, which lists structures, SMILES, molecular mass, calculated topological polar surface area, original indication, chemical name, mode of action (when available), listed target organism(s), compound names, resistome pools, and MMV compound number.

In agreement with the philosophy of Open Science, box recipients are expected to share their results in the public domain within 2 years. The data can be made available either via the ChEMBL database and/or by publication in a peer reviewed open access journal, with an acknowledgment to the source and supply of the compounds.^52^ The recipient should also acknowledge MMV, IVCC and BMS for assembling and supplying the box.

## Roll out of the GHPB Compound Set

**Figure 8:**
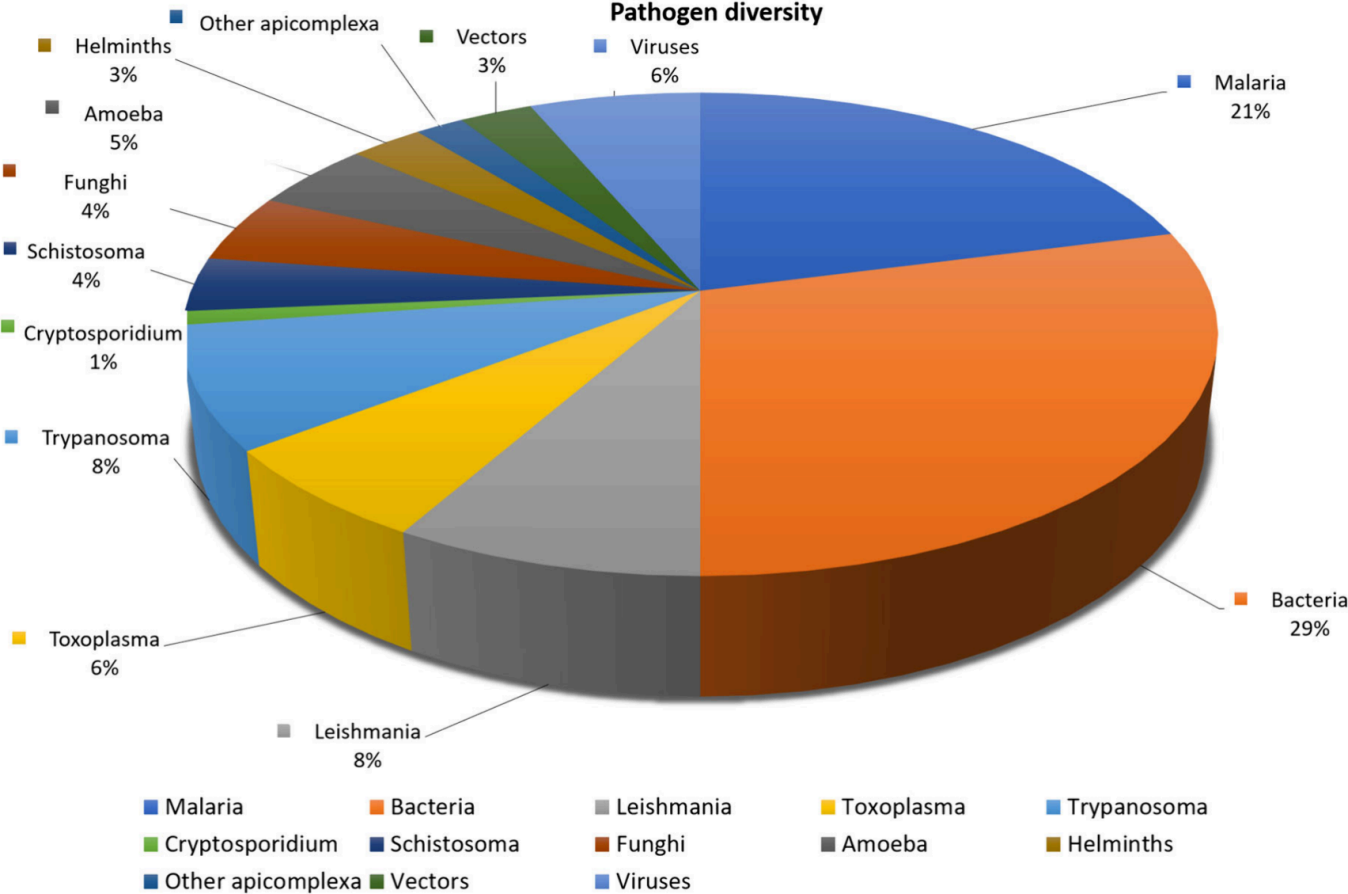
Pathogen diversity in the requests.

Since its launch in August 2022, the Global Health Priority Box was requested by over 120 researchers, indicating a great interest to screen high-quality compound collections. Moreover, over 25 researching institutions contacted MMV with request for resupply of compounds for hit confirmation and several promising hits were already identified.^54−56^ The availability of the GHPB also boosted the interest in other MMVOpen Access Boxes such as the Pandemic Response Box.^53^ These boxes have been sent for screening against various neglected pathogens and vectors; the vast majority of the requests were submitted for research in malaria and bacteria (Figure 8), with special attention to ESKAPE pathogens.^57^

By the end of Q2 2024, the GHPB was distributed to **30 different countries** around the globe, with over **50%** of them shipped to endemic countries located in Africa, Asia and South America.

**Figure 9:**
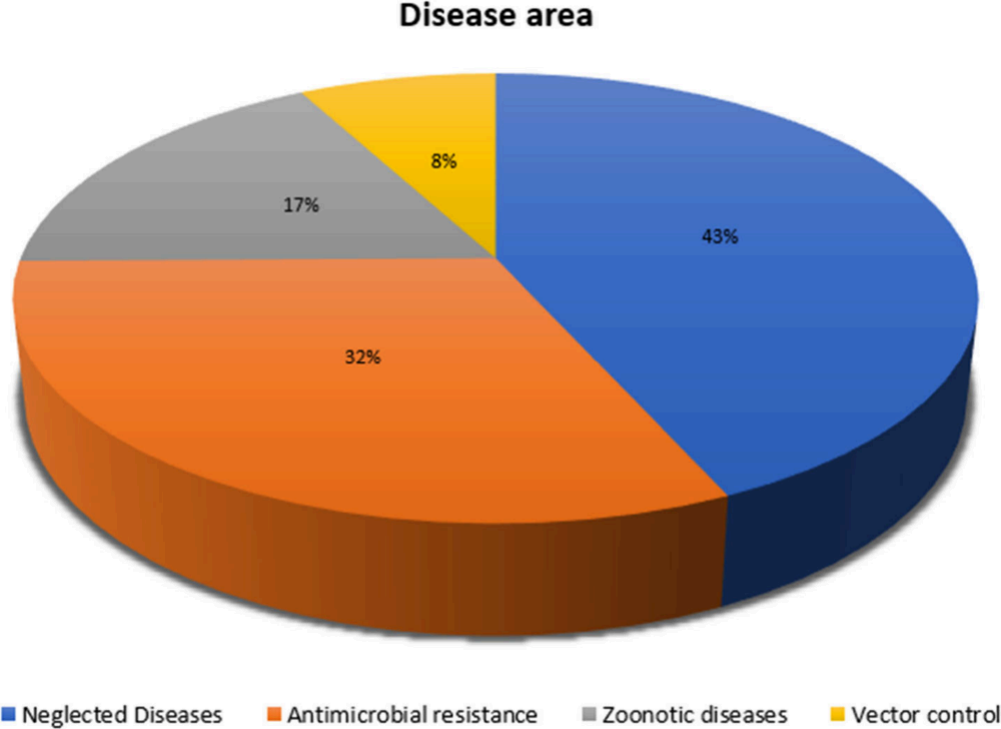
Number of requests by disease area.

Figure 9 illustrates the distribution based on disease area.

## Follow-up Guidelines

The Requestor shall not seek to obtain any intellectual property rights whatsoever in any of the compounds including, without limitation, any new formulations, uses (medical or otherwise), applications, or methods of administration thereof.

Researchers may refer to the following publications for the next steps once actives have been identified from the box:

- Actives from MMV Open Access Boxes? A suggested way forward.^4^
- A testing cascade to identify repurposed insecticides for next-generation vector control tools: screening a panel of chemistries with novel modes of action against a malaria vector.^58^

## Supporting Information Available

Box compounds with compound details (XLSX)
Footnotes to table of box compounds with compound details (PDF)
