# Supplementary material for: Global Health Priority Box—Proactive Pandemic Preparedness
Source: ACS Infect Dis. 2024 Nov 3;10(12):4030–9. doi: 10.1021/acsinfecdis.4c00700 (PMC11650653; doi:10.1021/acsinfecdis.4c00700)
Supplement: Supplementary file 1 — id4c00700_si_001.pdf [file id4c00700_si_001.pdf]

Supporting Information

Global Health Priority Box – proactive pandemic preparedness

Authors: Anna Adam[1], Dominique Besson[1*], Rob Bryant[4], Sarah Rees[2], Paul A. Willis[1], Jeremy N. Burrows[1], Rob Hooft van Huisjduijnen[1], Benoît Laleu[1], Larry Norton[2], Stacie Canan[3], Natalie Hawryluk[3], Dale Robinson[3], Mike Palmer[1], Kirandeep Kaur Samby[1£]

[1]MMV Medicines for Malaria Venture, 1215 Geneva, Switzerland
[2]Innovative Vector Control Consortium, L3 5QA Liverpool, United Kingdom
[3] Bristol Myers Squibb, CA 92121 San Diego , United States of America
[4]Brychem/Agranova, BR6 9AP Kent , United Kingdom

* Corresponding author

*Dominique Besson

bessond@mmv.org

[£]Senior author

Supporting Information

S1 List of Global Health Priority Box compounds with compound details

Acknowledgement

We thank Sir Simon Campbell, Mike Witty and Mary Mader (Molecular Innovation) for reviewing the drug resistant malaria set compounds. We thank Elizabeth Winzeler (University of California Sand Diego), Sergio Wittlin (Swiss Tropical and Public Health Institute), Marcus Lee (University of Dundee) for screening the malaria set compounds in resistant cell lines and Adele Lehane (The Australian National University) for fingerprint pH assay. We thank TCGLS group for synthesis of compounds.

Author information:
Kirandeep Kaur Samby – Former *MMV Medicines for Malaria Venture* contractor, *currently Johnson* & Johnson, Pvt Ltd, India
Anna Adam – MMV *Medicines for Malaria Venture, 1215 Geneva, Switzerland*
Rob Bryant – *Brychem/Agranova*, Kent BR6 9AP, United Kingdom
Sarah Rees – *Innovative Vector Control Consortium, Pembroke Pl, Liverpool L3 5QA, United Kingdom*
Paul A. Willis - *MMV Medicines for Malaria Venture, 1215 Geneva , Switzerland*
Jeremy N. Burrows – *MMV Medicines for Malaria Venture, 1215 Geneva , Switzerland*
Rob Hooft van Huisjduijnen – Former *MMV Medicines for Malaria Venture contractor, 1215 Geneva Switzerland*
Benoît Laleu – *MMV Medicines for Malaria Venture, 1215 Geneva , Switzerland*
***Dominique Besson** – *MMV Medicines for Malaria Venture, 1215 Geneva , Switzerland*
Larry Norton – *Innovative Vector Control Consortium, Pembroke Pl, Liverpool L3 5QA, United Kingdom*
Stacie Canan – Former *Bristol Myers Squibb, San Diego, CA, USA 92121*
Natalie Hawryluk- Former *Bristol Myers Squibb, San Diego, CA, USA 92121*
Dale Robinson – Former *Bristol Myers Squibb, San Diego, CA, USA 92121*
Mike Palmer -_Former MMV *Medicines for Malaria Venture contractor, 1215 Geneva Switzerland*

Author Contributions:

K.K.S., B.L., D.B., L.N., S.R., J.N.B. and P.A.W. designed and implemented the research plan described in the manuscript. K.K.S. supervised the synthesis of compounds. S.C., N.H. and D.R allocated and selected 3584 compounds for inclusion in BMS plate. K.K.S., D.B., A.A., B.L. and P.A.W. managed the launch and distribution of the Global Health Priority Box. A.A. and K.K.S. wrote the manuscript with input from all authors.

Funding
This work was supported, in whole or in part, by the Bill & Melinda Gates Foundation [INV-007155]. Under the grant conditions of the Foundation, a Creative Commons Attribution 4.0 Generic License has already been assigned to the Author Accepted Manuscript version that might arise from this submission.

Table S1

By default, compounds have been screened on MMV standard asexual blood stage assay[1], on standard panel of resistant cell lines[2], on yDHODH[3] assays, on basic cytotoxicity assay[4] and on pH fingerprint assay[5] to identify possible MoA and MoR. Some compounds were profiled in resistome pools[6] (MoR/MoA not identified* or MoR/MoA not identified**) containing resistant cell lines. *MoR/MoA not identified* indicates that the compounds do not show above mentioned resistance mechanisms or targets. Compounds with Pb liver or gametocytocidal activity were selected based on publications[7,8]

[1] *Pf* 3D7 LDH assay and cytotoxicity assay were carried out at **TCGLS, Kollkata**

[2,3,4] Profiling of compound in Dd2, Dd2 CARL-I1139K (Cyclic amine resistance locus (Pf CARL)), Dd2 PI4K-S1320L (Phosphatidylinositol-4-OH kinase (Pf PI4K)) and Dd2 ACS-A597V (Acetyl CoA synthetase (Pf ACS)) resistant cell lines, in scDHODH assay (*for Pf DHODH and ETC inhibitors*) as well as in cytotoxicity assay was carried out in **Elizabeth Winzeler lab at University of California San Diego (UCSD)**

[5] pH fingerprint assays were performed by **Adele Lehane group at Australian National University (ANU)**. It allows to possible identify Plasma membrane P-type ATPase (Pf ATP4), Formate-nitrite transporter (Pf FNT), the V-type H^+ ATPase, and the acid-loading Cl^- transport pathway as well as protonophores and possible inhibitors of PfHT or glycolysis inhibitors

[6] Profiling of compounds in resistome pools was done by **Marcus Lee group at Wellcome Sanger Institute**. Details of the pools are captured in the RESISTOME POOLS worksheet

[7] The compounds were Pb liver stage active compounds were selected from the publication *Open-source discovery of chemical leads for next-generation chemoprotective antimalarials*. Science. 2018 Dec 7;362(6419):eaat9446. https://doi.org/10.1126/science.aat9446 Antonova-Koch Y, Meister S, Abraham M et al.

[8] Compounds with gametocytocidal activity were selected from the publication *A high throughput screen for next generation leads targeting malaria parasite transmission*. Nat Commun 9, 3805 (2018). https://doi.org/10.1038/s41467-018-05777-2 Delves, M.J., Miguel-Blanco, C., Matthews, H. et al.

References:

1. About the Global Health Priority Box | Medicines for Malaria Venture.https://www.mmv.org/mmv-open/global-health-priority-box/about-global-health-priority-box (accessed 2024-07-03).

2. World Health Organization. 10 global health issues to track in 2021.https://www.who.int/news-room/spotlight/10-global-health-issues-to-track-in-2021 (accessed 2024-07-03).

3. Neglected tropical diseases -- GLOBAL. https://www.who.int/health-topics/neglected-tropical-diseases#tab=tab_2 (accessed 2024-07-03).

4. Samby K, Willis PA, Burrows JN, Laleu B, Webborn PJH. Actives from MMV Open Access Boxes? A suggested way forward. Kafsack BFC, editor. PLOS Pathogens. 2021 Apr 22;17(4):e1009384, DOI: 10.1371/journal.ppat.1009384

5. Trouiller P, Torreele E, Olliaro P, White N, Foster S, Wirth D, et al. Drugs for neglected diseases: a failure of the market and a public health failure? Tropical medicine & international health: TM & IH [Internet]. 2001 Nov 1;6(11):945–51, DOI: **10.1046/j.1365-3156.2001.00803.x**

6. World malaria report 2022. Geneva: World Health Organization; 2022. https://www.who.int/teams/global-malaria-programme/reports/world-malaria-report-2022 (accessed 2023-02-06)

7. World malaria report 2023. Geneva: World Health Organization; 2023. https://www.who.int/teams/global-malaria-programme/reports/world-malaria-report-2023 (accessed 2023-02-06)

8. Lindsay SW, Thomas MB, Kleinschmidt I. Threats to the effectiveness of insecticide-treated bednets for malaria control: thinking beyond insecticide resistance. The Lancet Global Health. 2021 Jun;9(9), DOI: 10.1016/S2214-109X(21)00216-3

9. Fairhurst RM. Understanding artemisinin-resistant malaria. Current Opinion in Infectious Diseases. 2015 Oct;28(5):417–25, DOI: 10.1097/QCO.0000000000000199

10. Fairhurst RM, Dondorp AM. Artemisinin-Resistant Plasmodium falciparum Malaria. Microbiology Spectrum. 2016 Jun 2;4(3), DOI: 10.1128/microbiolspec.EI10-0013-2016

11. Menard D, Dondorp A. Antimalarial Drug Resistance: A Threat to Malaria Elimination. Cold Spring Harbor Perspectives in Medicine. 2017 Jul 1;7(7), DOI: 10.1101/cshperspect.a025619

12. Wan-Yu Chu, Thomas P C Dorlo, Pyronaridine: a review of its clinical pharmacology in the treatment of malaria, *Journal of Antimicrobial Chemotherapy*, Volume 78, Issue 10, October 2023, Pages 2406–2418, DOI: 10.1093/jac/dkad260

13. Marwa K, Kapesa A, Baraka V, Konje E, Kidenya B, Mukonzo J, et al. Therapeutic efficacy of artemether-lumefantrine, artesunate-amodiaquine and dihydroartemisinin-piperaquine in the treatment of uncomplicated Plasmodium falciparum malaria in Sub-Saharan Africa: A systematic review and meta-analysis. Okell LC, editor. PLOS ONE. 2022 Mar 10;17(3):e0264339, DOI: 10.1371/journal.pone.0264339

14. Carter TE, Yared S, Gebresilassie A, Bonnell V, Damodaran L, Lopez K, et al. First detection of Anopheles stephensi Liston, 1901 (Diptera: culicidae) in Ethiopia using molecular and morphological approaches. Acta Tropica. 2018 Dec;188:180–6, DOI: 10.1016/j.actatropica.2018.09.001

15. Ali S, Samake JN, Spear J, Carter TE. Morphological identification and genetic characterization of Anopheles stephensi in Somaliland. Parasites & Vectors. 2022 Jul 8;15(1), DOI: 10.1186/s13071-022-05339-y

16. Ahmed A, Khogali R, Elnour M-AB, Nakao R, Salim B. Emergence of the invasive malaria vector Anopheles stephensi in Khartoum State, Central Sudan. Parasites & Vectors. 2021 Oct 2;14(1), DOI: 10.1186/s13071-021-05026-4

17. Ochomo EO, Milanoi S, Abong'o B, Onyango B, Muchoki M, Omoke D, et al. Molecular surveillance leads to the first detection of Anopheles stephensi in Kenya. 2023 Jan 21, DOI: 10.21203/rs.3.rs-2498485/v1

18. Tadesse FG, Ashine T, Teka H, Esayas E, Messenger LA, Chali W, Meerstein-Kessel L, Walker T, Wolde Behaksra S, Lanke K, Heutink R, Jeffries CL, Mekonnen DA, Hailemeskel E, Tebeje SK, Tafesse T, Gashaw A, Tsegaye T, Emiru T, Simon K, Bogale EA, Yohannes G, Kedir S, Shumie G, Sabir SA, Mumba P, Dengela D, Kolaczinski JH, Wilson A, Churcher TS, Chibsa S, Murphy M, Balkew M, Irish S, Drakeley C, Gadisa E, Bousema T. Anopheles stephensi Mosquitoes as Vectors of Plasmodium vivax and falciparum, Horn of Africa, 2019. Emerg Infect Dis. 2021 Feb;27(2):603-607, DOI: 10.3201/eid2702.200019

19. Enayati A, Hanafi-Bojd AA, Sedaghat MM, Zaim M, Hemingway J. Evolution of insecticide resistance and its mechanisms in Anopheles stephensi in the WHO Eastern Mediterranean Region. Malaria Journal. 2020 Jul 17;19(1), DOI: 10.1186/s12936-020-03335-0

20. Ashley EA, Phyo AP. Drugs in Development for Malaria. Drugs. 2018 Jun;78(9):861-879, DOI: 10.1007/s40265-018-0911-9

21. MMV's pipeline of antimalarial drugs | Medicines for Malaria Venture. https://www.mmv.org/research-development/mmvs-pipeline-antimalarial-drugs

(accessed 2023-02-06)

22. Shibeshi MA, Kifle ZD, Atnafie SA. Antimalarial Drug Resistance and Novel Targets for Antimalarial Drug Discovery. Infection and Drug Resistance. 2020 Nov;Volume 13:4047–60, DOI: 10.2147/IDR.S279433

23. Hovlid ML, Winzeler EA. Phenotypic Screens in Antimalarial Drug Discovery. Trends in Parasitology. 2016 Sep;32(9):697–707, DOI: 10.1016/j.pt.2016.04.014

24. Guiguemde WA, Shelat AA, Bouck D, Duffy S, Crowther GJ, Davis PH, et al. Chemical genetics of Plasmodium falciparum. Nature [Internet]. 2010 May;465(7296):311–5, DOI: 10.1038/nature09099

25. Plouffe D, Brinker A, McNamara C, Henson K, Kato N, Kuhen K, et al. In silico activity profiling reveals the mechanism of action of antimalarials discovered in a high-throughput screen. Proceedings of the National Academy of Sciences of the United States of America [Internet]. 2008 Jul 1 [cited 2022 Sep 23];105(26):9059–64, DOI: 10.1073/pnas.0802982105

26. Davis RL. Mechanism of Action and Target Identification: A Matter of Timing in Drug Discovery. iScience. 2020 Sep;23(9):101487, DOI: 10.1016/j.isci.2020.101487

27. Barber BE, Fernandez M, Patel HB, Barceló C, Woolley S, Patel H, et al. Safety, pharmacokinetics, and antimalarial activity of the novel triaminopyrimidine ZY-19489: a first-in-human, randomised, placebo-controlled, double-blind, single ascending dose study, pilot food-effect study, and volunteer infection study. Lancet Infectious Diseases. 2022 Jun 1;22(6):879–90, DOI: 10.1016/S1473-3099(21)00679-4

28. McCarthy JS, Özkan Yalkinoglu, Anand Odedra, Webster RK, Oeuvray C, Tappert A, et al. Safety, pharmacokinetics, and antimalarial activity of the novel plasmodium eukaryotic translation elongation factor 2 inhibitor M5717: a first-in-human, randomised, placebo-controlled, double-blind, single ascending dose study and volunteer infection study. Lancet Infectious Diseases. 2021 Dec 1;21(12):1713–24, DOI: 10.1016/S1473-3099(21)00252-8

29. Kuhen KL, Chatterjee AK, Rottmann M, Gagaring K, Borboa R, Buenviaje J, et al. KAF156 Is an Antimalarial Clinical Candidate with Potential for Use in Prophylaxis, Treatment, and Prevention of Disease Transmission. Antimicrobial Agents and Chemotherapy. 2014 Sep;58(9):5060–7, DOI: 10.1128/AAC.02727-13

30. World Health Organization. Vector-borne Diseases.Who.int. World Health Organization: WHO; 2020. https://www.who.int/news-room/fact-sheets/detail/vector-borne-diseases (accessed 2024-09-30)

31. Golding N, Wilson AL, Moyes CL, Cano J, Pigott DM, Velayudhan R, et al. Integrating vector control across diseases. BMC Medicine. 2015 Oct 1;13(1), DOI: 10.1186/s12916-

015-0491-4

32. Valenzuela JG, Aksoy S. Impact of vector biology research on old and emerging neglected tropical diseases. Walson JL, editor. PLOS Neglected Tropical Diseases. 2018 May 31;12(5):e0006365, DOI: 10.1371/journal.pntd.0006365

33. Schofield CJ, Jannin J, Salvatella R. The future of Chagas disease control. Trends in Parasitology.2006 Dec;22(12):583–8, DOI: 10.1016/j.pt.2006.09.011

34. Basáñez M-G, Pion SDS, Churcher TS, Breitling LP, Little MP, Boussinesq M. River Blindness: A Success Story under Threat? PLoS Medicine. 2006 Sep 26;3(9):e371, DOI: 10.1371/journal.pmed.0030371

35. Bhatt S, Weiss DJ, Cameron E, Bisanzio D, Mappin B, Dalrymple U, et al. The effect of malaria control on Plasmodium falciparum in Africa between 2000 and 2015. Nature [Internet]. 2015 Sep 16;526(7572):207–11, DOI: 10.1038/nature15535

36. Wondji CS, Coleman M, Kleinschmidt I, Mzilahowa T, Irving H, Ndula M, et al. Impact of pyrethroid resistance on operational malaria control in Malawi. Proceedings of the National Academy of Sciences. 2012 Nov;109(47):19063–70, DOI: 10.1073/pnas.1217229109

37. WHO. WHO publishes recommendations on two new types of insecticide-treated nets. 2023. https://www.who.int/news/item/14-03-2023-who-publishes-recommendations-on-two-new-types-of-insecticide-treated-nets (accessed 2023-04-06)

38. Accrombessi M, Cook J, Dangbenon E, Yovogan B, Akpovi H, Sovi A, et al. Efficacy of pyriproxyfen-pyrethroid long-lasting insecticidal nets (LLINs) and chlorfenapyr-pyrethroid LLINs compared with pyrethroid-only LLINs for malaria control in Benin: a cluster-randomised, superiority trial. The Lancet. 2023 Jan, DOI: 10.1016/S0140-6736(22)02319-4

39. van den Berg H, da Silva Bezerra HS, Al-Eryani S, Chanda E, Nagpal BN, Knox TB, et al. Recent trends in global insecticide use for disease vector control and potential implications for resistance management. Scientific Reports [Internet]. 2021 Dec 13;11(1):23867, DOI: 10.1038/s41598-021-03367-9

40. WHO. Neglected zoonotic tropical diseases. https://www.who.int/news-room/facts-in-pictures/detail/neglected-zoonotic-tropical-diseases (accessed 2023-02-06)

41. Taylor LH, Latham SM, woolhouse MEJ. Risk factors for human disease emergence. Woolhouse MEJ, Dye C, editors. Philosophical Transactions of the Royal Society of London Series B: Biological Sciences. 2001 Jul 29;356(1411):983–9, DOI: 10.1098/rstb.2001.0888

42. Woolhouse MEJ, Gowtage-Sequeria S. Host Range and Emerging and Reemerging

Pathogens. Emerging Infectious Diseases. 2005 Dec;11(12):1842–7, DOI: 10.3201/eid1112.050997

43. Wood JLN, Leach M, Waldman L, MacGregor H, Fooks AR, Jones KE, et al. A framework for the study of zoonotic disease emergence and its drivers: spillover of bat pathogens as a case study. Philosophical Transactions of the Royal Society B: Biological Sciences [Internet]. 2012 Oct 19;367(1604):2881–92, DOI: 10.1098/rstb.2012.0228

44. Holmes EC. COVID-19—lessons for zoonotic disease. Science. 2022 Mar 11;375(6585):1114–5, DOI: 10.1126/science.abn2222

45. AGRANOVA: Ag Chem Base.www.agranova.co.uk (accessed 2023-02-06)

46. Parasitipedia: Wellcome. www.parasitipedia.net (accessed 2023-02-06)

47. Yang T, Ottilie S, Istvan ES, Godinez-Macias KP, Lukens AK, Baragaña B, Campo B, Walpole C, Niles JC, Chibale K, Dechering KJ, Llinás M, Lee MCS, Kato N, Wyllie S, McNamara CW, Gamo FJ, Burrows J, Fidock DA, Goldberg DE, Gilbert IH, Wirth DF, Winzeler EA; Malaria Drug Accelerator Consortium. MalDA, Accelerating Malaria Drug Discovery. Trends Parasitol. 2021 Jun;37(6):493-507, DOI: 10.1016/j.pt.2021.01.009

48. Antonova-Koch Y, Meister S, Abraham M, Luth MR, Ottilie S, Lukens AK, et al. Open-source discovery of chemical leads for next-generation chemoprotective antimalarials. Science. 2018 Dec 7;362(6419), DOI: 10.1126/science.aat9446

49. Delves MJ, Miguel-Blanco C, Matthews H, Molina I, Ruecker A, Yahiya S, et al. A high throughput screen for next-generation leads targeting malaria parasite transmission. Nature Communications. 2018 Sep 18;9(1), DOI: 10.1038/s41467-018-05777-2

50. StarDrop: Small Molecule Drug Discovery & Data Visulization Software.Optibrium. https://optibrium.com/stardrop (accessed 2023-02-06)

51. Lindblom JCR, Zhang X, Lehane AM. A pH Fingerprint Assay to Identify Inhibitors of Multiple Validated and Potential Antimalarial Drug Targets. ACS Infect Dis. 2024 Mar 18, DOI: 10.1021/acsinfecdis.3c00588

52. Global Health Priority Box supporting information | Medicines for Malaria Venture . https://www.mmv.org/mmv-open/global-health-priority-box/global-health-priority-box-supporting-information (accessed 2023-02-06)

53. Bethencourt-Estrella CJ, Atteneri López-Arencibia, Lorenzo-Morales J, Piñero JE. Global Health Priority Box: Discovering Flucofuron as a Promising Antikinetoplastid Compound. Pharmaceuticals.2024 Apr 25 [cited 2024 Oct 4];17(5):554–4, DOI: 10.3390/ph17050554

54. Shanley HT, Taki AC, Byrne JJ, Nguyen N, Tim, Jabbar A, et al. A phenotypic screen of the Global Health Priority Box identifies an insecticide with anthelmintic activity. Parasites & Vectors. 2024 Mar 14;17(1), DOI: 10.1186/s13071-024-06183-y

55. Ma J, Eadie K, Schippers M, Fahal A, Laleu B, Verbon A, et al. Novel Compound MMV1804559 from the Global Health Priority Box Exhibits In Vitro and In Vivo Activity against Madurella mycetomatis. International Journal of Molecular Sciences [Internet]. 2024 Jun 5 [cited 2024 Oct 4];25(11):6227, DOI: **10.3390/ijms25116227**,

56. Samby K, Besson D, Dutta A, Patra B, Doy A, Glossop P, et al. The Pandemic Response Box–Accelerating Drug Discovery Efforts after Disease Outbreaks. ACS Infectious Diseases. 2022 Mar 14;8(4):713–20, DOI: 10.1021/acsinfecdis.1c00527

57. Santajit S, Indrawattana N. Mechanisms of Antimicrobial Resistance in ESKAPE Pathogens. BioMed Research International [Internet]. 2016;2016:1–8, DOI: 10.1155/2016/2475067

58. Lees R, Praulins G, Davies R, Brown F, Parsons G, White A, et al. A testing cascade to identify repurposed insecticides for next-generation vector control tools: screening a panel of chemistries with novel modes of action against a malaria vector. Gates Open Research. 2019 Jul 10;3:1464, DOI: 10.12688/gatesopenres.12957.2
